# Spatial distribution of stygobitic crustacean harpacticoids at the boundaries of groundwater habitat types in Europe

**DOI:** 10.1038/s41598-020-76018-0

**Published:** 2020-11-04

**Authors:** Mattia Iannella, Barbara Fiasca, Tiziana Di Lorenzo, Maurizio Biondi, Mattia Di Cicco, Diana M. P. Galassi

**Affiliations:** 1 Department of Life, Health and Environmental Sciences, University of L’Aquila, Via Vetoio, 67010 Coppito, L’Aquila Italy; 2 IRET-CNR, National Research Council, Florence, Italy

**Keywords:** Ecology, Zoology, Ecology, Environmental sciences

## Abstract

The distribution patterns of stygobitic crustacean harpacticoids at the boundaries of three different groundwater habitat types in Europe were analysed through a GIS proximity analysis and fitted to exponential models. The results showed that the highest frequency of occurrences was recorded in aquifers in consolidated rocks, followed by the aquifers in unconsolidated sediments and, finally, by the practically non-aquiferous rocks. The majority of the stygobitic harpacticoid species were not able to disperse across the boundaries between two adjacent habitats, with 66% of the species occurring in a single habitat type. The species were not evenly distributed, and 35–69% of them occurred from 2 to 6 km to the boundaries, depending on the adjacent habitat types. The distribution patterns were shaped by features extrinsic to the species, such as the hydrogeological properties of the aquifers, and by species’ intrinsic characteristics such as the preference for a given habitat type and dispersal abilities. Most boundaries between adjacent habitat types resulted to be “breaches”, that is transmissive borders for stygobitic harpacticoids, while others were “impermeable walls”, that is absorptive borders. Our results suggest that conservation measures of groundwater harpacticoids should consider how species are distributed within the different groundwater habitat types and at their boundaries to ensure the preservation of species metapopulations within habitat patches and beyond them.

## Introduction

The groundwater environment hosts a suite of species that complete their whole life cycle in the darkness. These species, called stygobites, are major providers of ecosystem services^1^ within ground water and may act as bioindicators of groundwater quality^2^ and connectivity between ground water and surface waters^3,4^. Stygobites are also ecosystem engineers as they actively modify the hydraulic properties of the aquifer sediments through burrowing and releasing faeces^5^. Stygobites are known to be more sensitive to pollutants and temperature increase than their surface-water relatives^6,7^. In this regard, Mammola et al.^8^ have recently drawn up a manifesto to bring to light the urgency to better manage quality and quantity of the groundwater resource and conserve its unique biodiversity, being these two aspects inextricably associated. For this reason, several conservation studies have aimed at preserving the stygobitic biodiversity at different spatial scales^7,9–13^.

Stygobitic invertebrates are an important part of the groundwater biodiversity^14^. Around 11–15% of the 17,000 freshwater animal species in Europe are stygobites, including some crustacean orders, families and genera, composed only by obligate groundwater-dwelling taxa. The Crustacea Copepoda are among the most abundant and species-rich group in ground water, rivalling only with the crustacean amphipods^15^. Copepods are ubiquitous in karst aquifers, in fissured aquifers in igneous rocks and in alluvial aquifers. A wide range of body morphologies are found across different habitat types, suggesting that copepods may be good indicators of habitat heterogeneity. They also show marked differences in microhabitat preferences^15–19^, normally requiring a mean living space of about 200 μm^20^ though they can move into smaller (< 60 μm) interstices as well^21^. Cyclopoida and Harpacticoida are the dominant meiofaunal orders in ground water, the former being represented by over 350 stygobitic species of the families Cyclopidae and Halicyclopidae, the latter by more than 700 groundwater species, primarily belonging to the families Canthocamptidae, Parastenocarididae and Ameiridae, followed by a few representatives of the Chappuisiidae, Miraciidae and Phyllognathopodidae^15^.

Harpacticoids are the group that best represent the dimensional category of the groundwater meiofauna in continental freshwaters^22^; they are small, with slender body forms, ubiquitous and highly diversified in ground water and associated environments, such as the hyporheic zone of streams and rivers^4^, the limnostygal in lakes, the epikarst^12^ and the benthic or inbenthic layers in the saturated karst^15,23^. Compared to the Copepoda Cyclopoida, with a few exceptions, the Harpacticoida show a lower attitude for dispersal because of their holobenthic lifestyle; they tend to spend the whole life cycle, from the first naupliar stage to the adult stage, in their native habitat^15^. In theory, harpacticoids, as well as other meiofaunal groups, may move from a groundwater habitat type to a contiguous one because their small size does not hinder the passage even in the finest sediments or the smallest fractures in rocks^24^.

Species distributions are shaped through the interplay of several factors which includes habitat selection, success of colonization, species persistence which, in turn, is determined on how well individuals discriminate between optimal and suboptimal habitat patches^25^. Habitat choice, which may evolve concomitantly with the ecological niche, comprises a suite of biological and environmental factors that prompt the occupancy of habitat patches where populations maximize their fitness. For instance, the boundaries between two adjacent groundwater habitats may act as either “impermeable walls” (absorptive boundaries^26^), especially for species with narrow niche breadths or, alternatively, as “breaches” (transitional habitats, transmissive boundaries^26^). The boundaries between groundwater habitat types may be considered both tangible (structures that can be identified in nature) and relict (arisen from forces no longer operating in the area under study)^26,27^.

In conclusion, the potential of a species to live at a given boundary between different groundwater habitats depends on the nature of the border itself, on the habitats on either side and on the attitude of the species^27–29^. In order to manage and conserve groundwater biodiversity, it is essential to know how stygobitic species are distributed within the different groundwater habitat types^30^ and at the boundaries between them. In fact, the risk of endangering does not rely upon only biological aspects, but also on topological aspects of the landscapes, such as size, connectivity and the existence of corridors^31^. However, this aspect has been poorly studied to date and represents one of the main issues in groundwater ecology^13,30,32^. To this end, we examined the effect of boundaries between pairs of groundwater habitat types in terms of frequency of occurrences of the stygobitic harpacticoids in Europe, based on the effective records available for all the species at the continental scale. We used the groundwater habitat types mapped by Cornu et al.^30^ as functional units to perform GIS proximity analyses, so as to gather all the possible patterns of spatial occupancy in terms of habitat variability. GIS analyses are useful tools, both alone or coupled with other spatial techniques, to assess and quantify biodiversity-related topics (e.g.^33–35^). Regression analyses were also performed to define the influence of the distance (in km) from the boundaries on the frequency of occurrences of the groundwater harpacticoids. We aimed at offering new insights (1) to explore the patterns of frequency of species occurrences within each habitat type and at their respective boundaries, given that boundaries between different groundwater habitat types may be ecologically different to each other; (2) to assess which species are able to cross the boundaries, and which are not; (3) to evaluate the role of the boundaries in preserving metapopulations of the groundwater harpacticoid species.

## Results

### Records of stygobitic harpacticoids across groundwater habitat types of Europe

**Figure 1:**
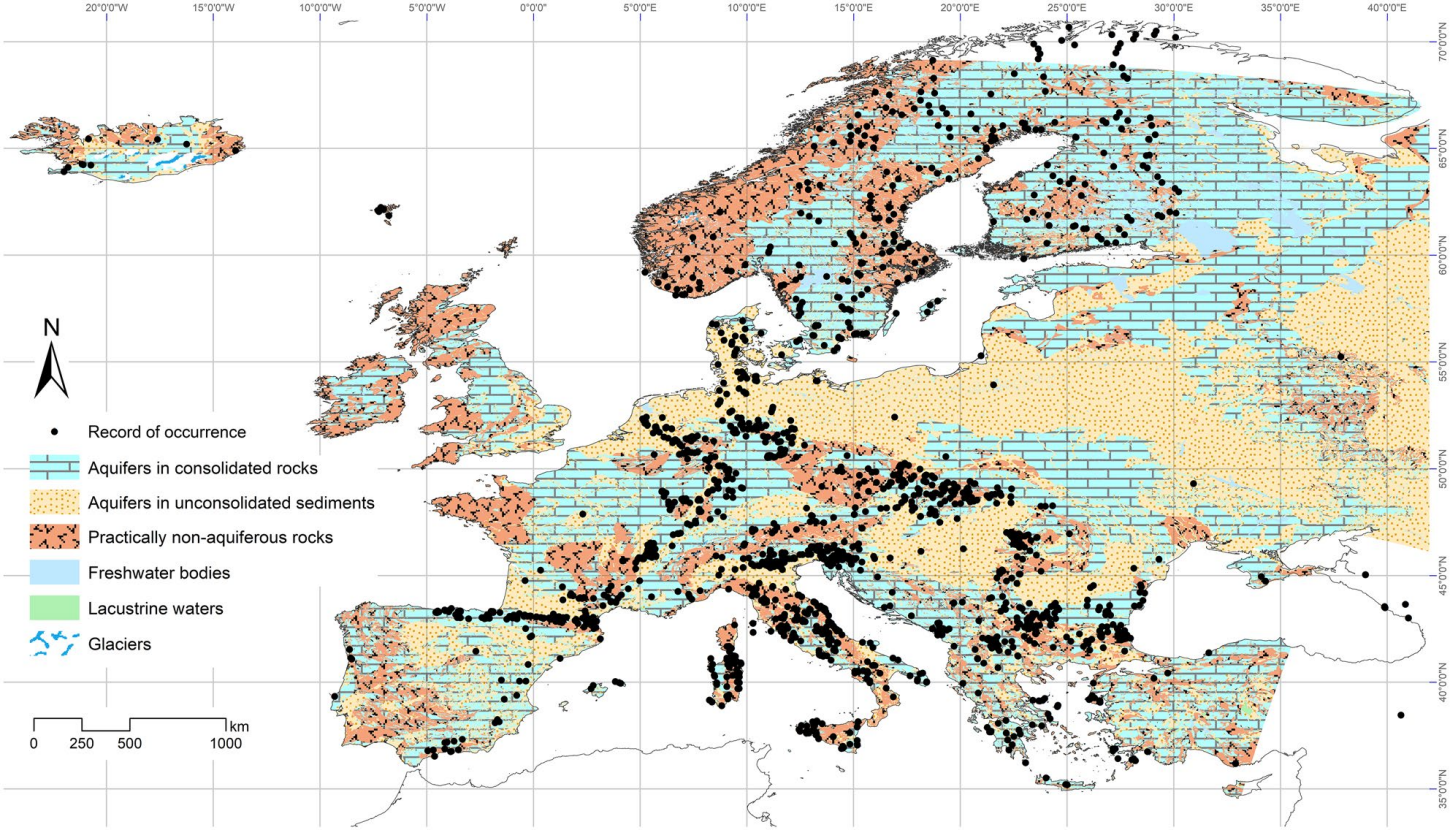
Distribution of the 3248 occurrence records (black dots) of the European stygobitic harpacticoid species in the three groundwater habitat types^30^ (freely available to: https://atlas.freshwaterbiodiversity.eu/index.php/explore/item/66-groundwater-habitats-europe-atlasapp): aquifers in consolidated rocks (CONS), aquifers in unconsolidated sediments (UNCONS), and practically non-aquiferous rocks (NonAQ). Features such as “glaciers”, “lacustrine waters”, and “freshwater bodies” were represented to enhance readability (map generated by ArcMap 10.0^55^; https://www.esri.com).

A total of 12,867 sampling sites for 21,700 records of occurrence of stygobitic crustaceans was analysed^36^ of which 2131 sampling sites hosted harpacticoids with 3248 records of occurrence A total of 408 stygobitic harpacticoid species/subspecies, distributed in 7 families and 42 genera, were recorded from groundwaters of Europe^13^ (Supplementary Table S1: List species_subspecies) with the following distribution across groundwater subhabitats: 29% of the records were from the interstitial environment of streams and rivers, 28% from caves, 21% from boreholes, 11% from springs, 2% from limnostygal; for the remaining 9% of the records the subhabitat was not defined. The distribution of the 408 stygobitic harpacticoids in the context of groundwater habitat types of Europe^30^, is represented in Fig. 1. Species’ occurrence records showed higher density south to the 45th parallel^13,36,37^ (Fig. 1).

A total of 358 out of 408 species were recorded at a distance < 20 km from any surrounding boundary. Moreover, stygobitic harpacticoids were not evenly distributed among groundwater habitat types: 147 species were exclusive of CONS, 43 of UNCONS, and 45 of NonAQ. The remaining 123 species occurred in more than one groundwater habitat type.

### GIS proximity analysis

The proximity analysis showed a good fit (> 73%) with the exponential model for all the six groups: R^2^ = 0.871 for CONS from CONS/UNCONS; R^2^ = 0.737 for CONS from CONS/NonAQ; R^2^ = 0.979 for UNCONS from CONS/UNCONS; R^2^ = 0.811 for UNCONS from UNCONS/NonAQ; R^2^ = 0.970 for NonAQ from CONS/NonAQ, and R^2^ = 0.876 for NonAQ from UNCONS/NonAQ. Further, the normal probability plots of fitted residuals showed a linear trend (Supplementary Figure S1), thus confirming the validity of the statistical assumptions for each regression.

All six groups showed a normal distribution (K–S test, *p* = 0.05), with CONS from CONS/UNCONS *p* = 0.598, CONS from CONS/NonAQ *p* = 0.608, UNCONS from CONS/UNCONS *p* = 0.162, UNCONS from UNCONS/NonAQ *p* = 0.278, NonAQ from UNCONS/NonAQ *p* = 0.211, and NonAQ from CONS/NonAQ *p* = 0.148. The Levene test (Sq-Dev) resulted in homogeneity of variance (F = 1.318, *p* = 0.261). When assessing differences among the six groups, the one-way ANOVA showed high power (0.991, *p* = 0.05) and statistical difference among groups (F_(5,120)_ = 5.692, *p* = 9.5 × 10^–5^). The Holm-Bonferroni test highlighted statistical differences between the means of four different pairs of groups, namely: UNCONS from CONS/UNCONS and CONS from CONS/UNCONS (*p* = 0.00136, α_H-Bcorr_ = 0.00417), UNCONS from UNCONS/NonAQ and CONS from CONS/UNCONS (*p* = 4.47 × 10^–4^, α_H-Bcorr_ = 0.00385), NonAQ from CONS/NonAQ and CONS from CONS/UNCONS (*p* = 6.20 × 10^–5^, α_H-Bcorr_ = 0.00357) and NonAQ from UNCONS/NonAQ and CONS from CONS/UNCONS (*p* = 1.81 × 10^–5^, α_H-Bcorr_ = 0.00333).

**Figure 2:**
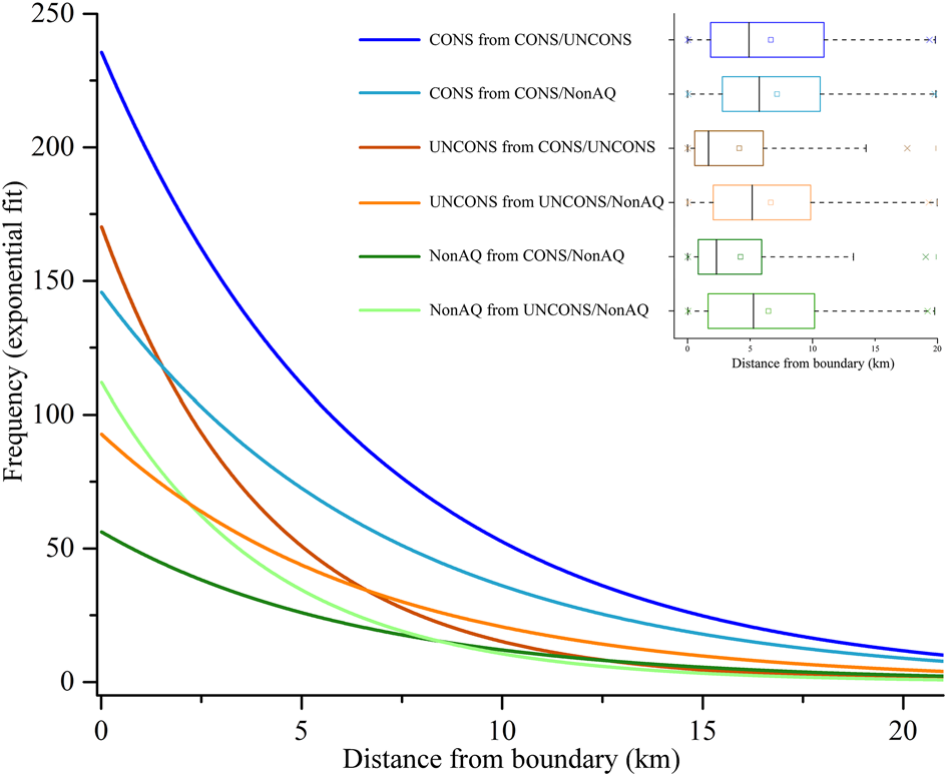
Trends of frequency of occurrences (exponential fit) of the stygobitic harpacticoid copepods at the boundaries between each group of groundwater habitat types; upper right: boxplots of the same set of data, representing median (black line within the boxplot), mean (square), and min/max values (whiskers) (*CONS* aquifers in consolidated rocks, *UNCONS* aquifers in unconsolidated sediments, *NonAQ* practically non-aquiferous rocks).

In all six groups, the highest frequency of occurrences was found close to the boundaries, with an exponential decrease as a function of the distance from the boundary (Fig. 2). This pattern was particularly evident in the CONS from CONS/UNCONS, and in the UNCONS from CONS/UNCONS groups, within which the species occurrence records for CONS from CONS/UNCONS showed steepest decrease in function of the distance from the boundary (Fig. 2). Records from both CONS and UNCONS at the boundary with NonAQ showed a similar trend of slow frequency decrease (Fig. 2). Species’ occurrences in NonAQ had the lowest frequencies at the boundary with CONS (Fig. 2) and the frequency of occurrences decreased very slowly. However, the fitted model of NonAQ from CONS/NonAQ boundary was significantly different (*p* = 6.20 × 10^–5^) from that of CONS from CONS/UNCONS only. The frequency of occurrences in NonAQ at the boundary with UNCONS was higher, with a steep frequency decrease as the distance from the boundary increased. However, the fitted model of NonAQ from UNCONS/NonAQ boundary was significantly different (*p* = 1.81 × 10^–5^) from that of CONS from CONS/UNCONS only. The median frequency of species’ occurrences in UNCONS from CONS/UNCONS and NonAQ from CONS/NonAQ was found at about 2 km from the boundary; the median frequency was found at about 6 km in CONS at the boundary to NonAQ and at 5 km for all other groups (Fig. 2, upper-right panel).

Cumulatively, the aquifers in consolidated rocks were the most species rich, with 69% and 35% of the total number of species recorded at least once within 5 and 6 km from the boundary with aquifers in unconsolidated sediments and practically non-aquiferous rocks, respectively (Table 1; Supplementary Table S1). In all the remaining groups, the number of species recorded within the distance range of MFO (median frequency of occurrences) varied from 48 to 55% of the total number of species (Table 1; Supplementary Table S1).

**Table 1:** Total number of stygobitic species occurring in each of the six groups (Supplementary Table S1); number of stygobitic species occurring from the boundary to the distance within which the median frequency of occurrences (MFO) was observed in each group (Fig. 2, upper right panel); number of species (in %) occurring within the distance range of MFO in brackets.

Group	Total N. species	N. species within the distance range of MFO	Distance range of MFO (km)
CONS from CONS/UNCONS	236	163 (69%)	5
CONS from CONS/NonAQ	162	56 (35%)	6
UNCONS from CONS/UNCONS	139	76 (55%)	2
UNCONS from UNCONS/NonAQ	121	60 (50%)	5
NonAQ from CONS/NonAQ	129	64 (50%)	2
NonAQ from UNCONS/NonAQ	88	42 (48%)	5

Stygobitic harpacticoid species had a different distribution pattern at the boundaries between groundwater habitat types, depending on the groundwater habitat types analysed. Some genera and species showed occurrences mainly in one type of groundwater habitat and at a small distance from the boundary. For example, the harpacticoid genus *Antrocamptus* occurred mostly in CONS. *Antrocamptus catherinae* occurred in CONS, mainly at a distance of 300 m from the boundary with UNCONS, and it was only exceptionally recorded in NonAQ at a distance of 600 m from the boundary, where this species lives in the thin layer of sediments overlying the almost impermeable NonAQ. *Antrocamptus chappuisi* mirrored the behavior of *A. catherinae*, only occasionally occurring in NonAQ (at 600 m from the boundary)*. Antrocamptus coffaiti* was exclusive to aquifers in consolidated rocks, recorded only from the saturated karst and showing a relatively broad distribution within CONS, never occurring in other groundwater habitat types. *Antrocamptus longifurcatus* and *Antrocamptus stygius* were tightly located at the boundary CONS/NonAQ, with low frequency of occurrences. A similar pattern was observed for the species of the canthocamptid genus *Lessinocamptus*; they were all confined to CONS, within the first 5 km from the boundary CONS/UNCONS. Finally, the ameirid *Nitocrella gracilis* was distributed in CONS with all records distributed within 2.5 km from the boundary CONS/UNCONS, never occurring in UNCONS; the species was recorded from NonAQ only sporadically, at 500 m beyond the boundary CONS/NonAQ.

Other species that were also found in CONS had a less restricted distribution and were not confined to areas within a few kilometers of the borders of the groundwater habitats. For instance, *Nitocrella psammophila* and *Elaphoidella elaphoides* showed high frequency of occurrences across large areas in CONS. In addition, *E. elaphoides* was frequently found in UNCONS as well, though confined to the first 500 m from the boundary. *N. psammophila* was widely distributed in CONS within 4–7 km of the CONS/UNCONS border. The parastenocaridid *Parastenocaris glacialis* was widely distributed in CONS, mainly within 2 km from the boundary CONS/UNCONS. *P. glacialis* and *Proserpinicaris phyllura* were also very frequently found in NonAQ, especially from Sweden and Norway and, to a less extent, from Finland; actually, the prevailing environment to which their occurrences are associated was the tiny sediment layers of small streams overlying NonAQ. Stygobitic species of the canthocamptid *Bryocamptus* and *Ceuthonectes* were widely distributed in CONS, with high frequency of occurrences at the CONS side of the boundary CONS/UNCONS. In the UNCONS, at the CONS/UNCONS boundary, *Parastenocaris gertrudae*, *P. glacialis* and *P. phyllura* showed a tendency to set close to the boundary.

The species showing high frequency of occurrences in CONS, at the boundary CONS/UNCONS, were widely distributed within their own habitat type, and only occasionally their records were clumped. Exceptions were found for genera and species which were almost exclusive to CONS, which showed a grouped distribution of their records.

In CONS, at the boundary CONS/NonAQ, species’ records were stratified. In fact, some typical karstic species of the family Canthocamptidae (e.g. *Bryocamptus pyrenaicus*, *Bryocamptus balcanicus*, *Bryocamptus dacicus*, *Ceuthonectes gallicus* and *Ceuthonectes serbicus*) occurred within 4 km of the boundary; the family Parastenocarididae occurred most frequently in terms of records of genera and species between 4 and 9 km (e.g. *P. glacialis*, *P. phyllura*, *Horstkurtcaris nolli nolli*); the family Ameiridae was mostly recorded at distances greater than 9 km from the border (e.g. *N. psammophila*, *Nitocrella stammeri*, *Parapseudoleptomesochra subterranea*), and the family Chappuisiidae was mostly recorded at a distance of 15–20 km from the border.

In UNCONS, at the CONS/UNCONS boundary, many species showed the highest frequency of occurrences very close to the border. For instance, the species belonging to the families Canthocamptidae, Parastenocarididae and Ameiridae all had the highest frequency of occurrences within the first 2 km of the boundary. However, some species of Parastenocarididae and Ameiridae were widely distributed in UNCONS, while some species of Chappuisiidae were distributed mainly from 12 to 17 km from the CONS/UNCONS boundary on the CONS side.

In UNCONS, at the boundary UNCONS/NonAQ, species’ frequency of occurrences stratified spatially, with species belonging to Canthocamptidae showing the highest frequency of occurrences within 2 km of the boundary (species of the genera *Bryocamptus* and *Elaphoidella*, most of them shared also with CONS). From 2 to 6 km from the same boundary, a dominance of records of the Parastenocarididae was observed with members of *Parastenocaris*, *Minutacaris*, *Italicocaris*, *Horstkurtcaris*; from 6 to 9 km from the boundary only the Ameiridae were recorded, and the occurrences of the Chappuisiidae were the most distant from the boundary, from 13 to 18 km from the border.

In NonAQ, at the boundary CONS/NonAQ, most species’ records were close to the border. The records closest to the border were represented by Canthocamptidae, followed by Parastenocarididae and Ameiridae mixed together, and mainly recorded 2 km from the border.

In NonAQ, at the boundary UNCONS/NonAQ, the records of the Canthocamptidae were the closest to the boundary, with low species richness; the Parastenocarididae was the sole family recorded from less than 1 up to 5 km from the border, and the Ameiridae occurred at more than 5 km from the boundary, mixed with a few records of Parastenocarididae and Canthocamptidae. All records and distances from the boundaries between groundwater habitat types are listed in Supplementary Table S1.

At a smaller spatial scale, in the Pyrenean area, the trend of frequency of occurrences of the stygobitic harpacticoid copepods at the boundaries between each pair of groundwater habitat types mirrored the ones obtained at the broad European scale (Supplementary Figure S2). The data deriving from a smaller scale approach (Supplementary Figure S2) were not normally distributed (K–S test, *p* = 0.05, Supplementary File S1); thus, a Kruskal–Wallis test was performed, resulting, as in the European-scale analysis, in significant differences among the six groups (χ^2^ = 60.72, *df* = 5, *p* = 8.6 ×10^–12^). Further, the post-hoc Wilcoxon Rank Sum test highlighted significant differences in nine pairs of groups (Supplementary File S1).

## Discussion

Efforts to shed light on patterns and processes that shape groundwater biodiversity on broad spatial scales have been numerous. For instance, the pioneering study of Deharveng et al.^10^ on the distribution of the stygodiversity in gridded maps across five southern-European countries laid the foundations for biodiversity conservation of groundwater species. Stein et al.^38^ proposed the idea of European stygoregions that each may cover up to 100,000 km^2^. According to Stoch and Galassi^37^, the high species replacement observed across 100 km among regional aquifers in the Alpine arc within the same stygoregion, suggests a more restricted extension of the European stygoregions. Zagmajster et al.^36^ highlighted that stygobitic crustacean assemblages across Europe are almost entirely replaced within a distance of less than 500 km, but in this case, there was the constraint of the cell size (500 km^2^), and the downscaled interpretation remains challenging. Finally, Iannella et al.^13^ identified eight hotspots for the conservation of the European stygobitic harpacticoids at the groundwater habitat scale, located predominantly south to the 45th parallel, in line with the results of previous studies concerning groundwater invertebrate species. The operational units used by Iannella et al.^13^ were the same groundwater habitat types that were also used in this study. The adoption of three discrete groundwater habitat types for assessing where groundwater harpacticoids are distributed may be useful for conservation purposes under the rules of the Habitats Directive^39^ because the species can be adequately protected by protecting the habitats where they live. The present study expanded further in this direction and aimed at establishing how stygobitic harpacticoid species are distributed among different habitat types, with particular reference to the areas where the habitats are in contact (i.e. at the boundaries).

The results of the analyses carried out in this study showed that: (1) the aquifers in consolidated rocks were generally the richest in species, followed by the aquifers in unconsolidated sediments and, finally, by the practically non-aquiferous rocks; (2) more than half of the European stygobitic harpacticoid species showed limited dispersal across the boundaries, with 66% of the species occurring in a single habitat type; (3) the species were not evenly distributed in the different habitats, and were typically clumped near the boundaries (from 35 to 69% of the species, depending on the adjacent habitat types, in a strip from 2 to 6 km of the boundary). These results are in line with the considerations of Potts et al.^28^ who highlighted that, at broad spatial scales, animals cannot explore the whole landscape before setting in a habitat different from the native one. They are limited by their dispersal abilities, and this consistently applies to the small-sized stygobitic harpacticoids, which tend to remain in the place where they were born. This behavior may depend on the width of the niche breadth and on the mobility of the individuals. Both factors dictate the distance to which a population of a given species may disperse in the “stygoscape” and the species ability of crossing the boundaries among groundwater habitat types.

The potential of a species of crossing the border between groundwater habitats also depends on its spatial niche and how this is constructed and on the characteristics of the subhabitats in the contiguous habitat types. It should be noted that the subhabitats of a given groundwater habitat type may show environmental features which mirror those of a different groundwater habitat type. For example, in caves (CONS), some species live in the sediments present in the dripping pools. In NonAQ, other species reside in the tiny sediment layers overlying igneous rocks. These sediments mirror the microhabitats which prevail in UNCONS.

The intrinsic properties of the boundaries, depending on the nature of the adjacent habitat types, cannot be neglected. For example, the distribution of the occurrences of stygobitic harpacticoid species in the CONS habitat type at the boundary with UNCONS was significantly different from that observed in other habitat pairs, being characterized by the highest frequency of occurrences in the very first kilometres from the boundary. This pattern was due to the fact that aquifers in consolidated rocks, such as karst aquifers, frequently have their groundwater outlets naturally located at the contact with less permeable rocks. This favours the outflow of ground water and its resident fauna at the boundary and, also, beyond it. In fact, despite the majority of species occurring in aquifers in consolidated rocks were confined to this habitat, some other species were able to cross the boundary, especially with aquifers in unconsolidated sediments, and some other species—though fewer—were able to cross the border with NonAQ. Indeed, the small fractures in igneous rocks filled by ground water are the only microhabitats where “karstic species” may eventually disperse and settle in a more or less stable way. The species which showed the highest attitude to cross the boundaries at the CONS side of CONS/UNCONS boundary were *N. psammophila*, *P. glacialis*, *F. fontinalis fontinalis*, *P. phyllura*, *H. nolli nolli*, *E. elaphoides*, and *C. serbicus*.

Potential for dispersal seemed to be relatively high for some species living in CONS aquifers, which apparently yield more species to the neighbouring UNCONS aquifers. For example, the stygobitic species of the canthocamptid genus *Bryocamptus* (e.g. *B. dentatus*, *B. pyrenaicus*, *B. unisaetosus*), which occurred mainly at 1 km from the border, were able to cross the CONS/UNCONS boundary into the UNCONS. However, it is not the proximity of the occurrences to the boundary that favours the dispersion of a species from one type of habitat to another, though 5 km seemed to represent the distance from which the potential for dispersal of ‘karstic species’ steeply decreases. For example, members of the canthocamptid *Antrocamptus* and *Lessinocamptus*, despite occurring close (< 5 km) to the CONS/UNCONS boundary, remained confined to CONS, suggesting that other factors, such as microhabitat specificity^40^, a narrow niche breadth, and low potential for dispersal come into play in explaining what happens at the boundaries between different habitat types.

In the UNCONS, stygobitic harpacticoid species living in the interstitial voids among the sediment particles of streams and rivers, even if sometimes can disperse over several kilometers in the hyporheic zone or in an alluvial aquifer, tightly adhere to the sediment particles^15^. Hence, the migration from the hyporheic zone to the underlying aquifer in consolidated rocks is practically unfeasible. Indeed, the ectinosomatid species *Pseudectinosoma janineae* and the ameirid *Nitocrellopsis rouchi*, both known from the alluvial plain of the Rhône River were recorded from the saturated alluvial aquifer and the hyporheic zone. These species expressed a high preference for the UNCONS and a poor ability to disperse across the border between different habitat types. This observation is in line with what observed by Rouch^41^ in the Baget karstic system (Pyrenees) and its associated alluvial aquifer where 22 and 21 species were found, respectively, with only 12 species in common. Similarly, in the Dorvan karstic system (French Jura) and the adjacent alluvial aquifer of the Albarine River, 22 and 21 species were recorded, respectively, sharing only 8 species^42^. Stygobitic harpacticoids from aquifers in unconsolidated sediments only rarely dispersed into aquifers in consolidated rocks but there are exceptions. For instance, *P. phyllura*, whose preferred habitats are the hyporheic zone and the saturated aquifers in UNCONS, occurred in CONS and in NonAQ as well, with a broad distribution in the three groundwater habitat types, and occurring either close to the boundaries or widely dispersed within each groundwater habitat type. Similarly, *P. glacialis* is known from Europe with 183 records from UNCONS, CONS and NonAQ. Most records were from the hyporheic zone of streams and rivers, and rarely from the saturated karst.

Based on the results of this study, the NonAQ habitat type is the one characterized by the lowest number of occurrences of stygobitic harpacticoids. This pattern is partly due to the fact that practically non-aquiferous rocks do not retain enough ground water to be considered suitable habitats for stygobitic species.

The dispersal of stygobitic harpacticoids from NonAQ to CONS (but also from UNCONS to CONS, as previously mentioned) is uncommon, despite some species may disperse across NonAQ, which, to a certain degree, mirrors the fractured epikarst.

Sampling gaps might be claimed to explain the observed distribution of stygobitic harpacticoids among the three groundwater habitat types and at their boundaries. Nevertheless, the patterns observed at continental and regional scales cannot be explained solely by unbalanced sampling effort, also considering that our results are based on a consistent set of records and matched the distribution patterns of European groundwater crustaceans based on previously published information e.g.^13,32,36,37^. The results of this study suggest that stygobitic harpacticoid species could have a complex pattern of distribution in areas designated as hotspots of species richness^13^ due to multiple factors involving the niche breadth, the species attitude to disperse and the hydrogeological conditions of the habitat itself.

The proximity analysis indicated the relevance of the boundaries between groundwater habitat types in the conservation of groundwater biodiversity. Stygobitic harpacticoid species showed different frequency of occurrences at the borders between different pairs of habitat types, in part for reasons extrinsic to the species themselves, such as the hydrogeological properties of the three aquifer types, and partly for the adaptive characteristics of the species to particular habitat types, together with typically low attitudes for dispersal. Most boundaries between two groundwater habitat types resulted to be “breaches”, that is transmissive edges for stygobitic harpacticoids, while others were “walls”, that is absorptive edges and, in these cases, species were confined to one habitat type only.

The groundwater fauna is imperilled due to anthropogenic disturbance. Stygobites are particularly vulnerable due to their (typically) longer life span, low reproductive rate, low population abundances, short-range distribution (< 1–100 km^2^), all features that dramatically lower the resilience of groundwater communities^8,18,43^.

This study highlighted that conservation measures should ensure the preservation of metapopulations^44–46^ of the stygobitic harpacticoid species in the groundwater habitat types, and beyond, at the boundaries between them^13^, thus ensuring species’ survival^13^ if environmental changes occur that do not allow the suitability of one or more habitats for longer^44–46^.

Further, the uneven distribution of species makes the delimitation of biodiversity hotspots temporary, because what is now a hotspot of species richness can become a coldspot^44^. Under a conservation perspective, many hotspots of groundwater biodiversity embrace more than one habitat type^13^; thus, a collective awareness of the need to move beyond the biodiversity hotspot concept, as primarily defined by Myers et al.^47^ is needed.

## Methods

### Study area and dataset

The study area embraced the European continent, main islands included (longitude min = −31.3, longitude max = 65.2; latitude min = 27.6, latitude max = 69.2; decimal degrees). The area is a mosaic of 61,275 patches, each representing one out of the three groundwater habitat types identified upon the criteria of the groundwater flow type^30^, namely: (1) aquifers in consolidated rocks (CONS); (2) aquifers in unconsolidated sediments (UNCONS), and (3) practically non-aquiferous rocks (NonAQ). The main environmental features of the three groundwater habitat types are related to hydrogeology and retrieved from the International Hydrogeological Map of Europe (IHME; scale: 1:500,000) which represents the most comprehensive source of hydrogeological information at the European scale^30^. The groundwater flow is intergranular in UNCONS, it occurs mainly in fissures and fractures in CONS (though some intergranular flow may also occur), and is almost inexistent in NonAQ. Permeability is mainly high in CONS, high-moderate in UNCONS and very low in NonAQ. Finally, pore size is mainly small in CONS, large in UNCONS and very small in NonAQ.

Each groundwater habitat type includes different subhabitats and microhabitats that can be colonized by different groundwater assemblages^43^. CONS includes, for example, dripping pools, puddles, subterranean streams and lakes in caves, and groundwater-fed springs in the unsaturated and the saturated karst^12,17,19,41,42,48–50^. UNCONS comprises alluvial aquifers and hyporheic zones and, finally, NonAQ is mainly described by small fractures in igneous rocks filled with ground water. The occurrences of the stygobitic harpacticoids in each patch were retrieved from Limnofauna Europaea^51^, Stygofauna Mundi^52^, the European PASCALIS database^10^, the Hypogean Crustacea Recording Scheme^53^, the Checklist of the Italian Fauna^54^, personal bibliographic collections and unpublished data (D.M.P.G.). For a few species, distribution maps from the literature were scanned and georectified and the coordinates of occurrence points were computed in ArcMap 10.0 software^55^. Synonymies and incorrect species names were improved and only accepted names were included in the dataset. Undescribed species which were recognized as new to science by taxonomists (D.M.P.G.) were included in the dataset. The list of the stygobitic harpacticoid species is provided in the Supplementary Table S1 (List species_subspecies).

### Spatial and statistical analyses

**Figure 3:**
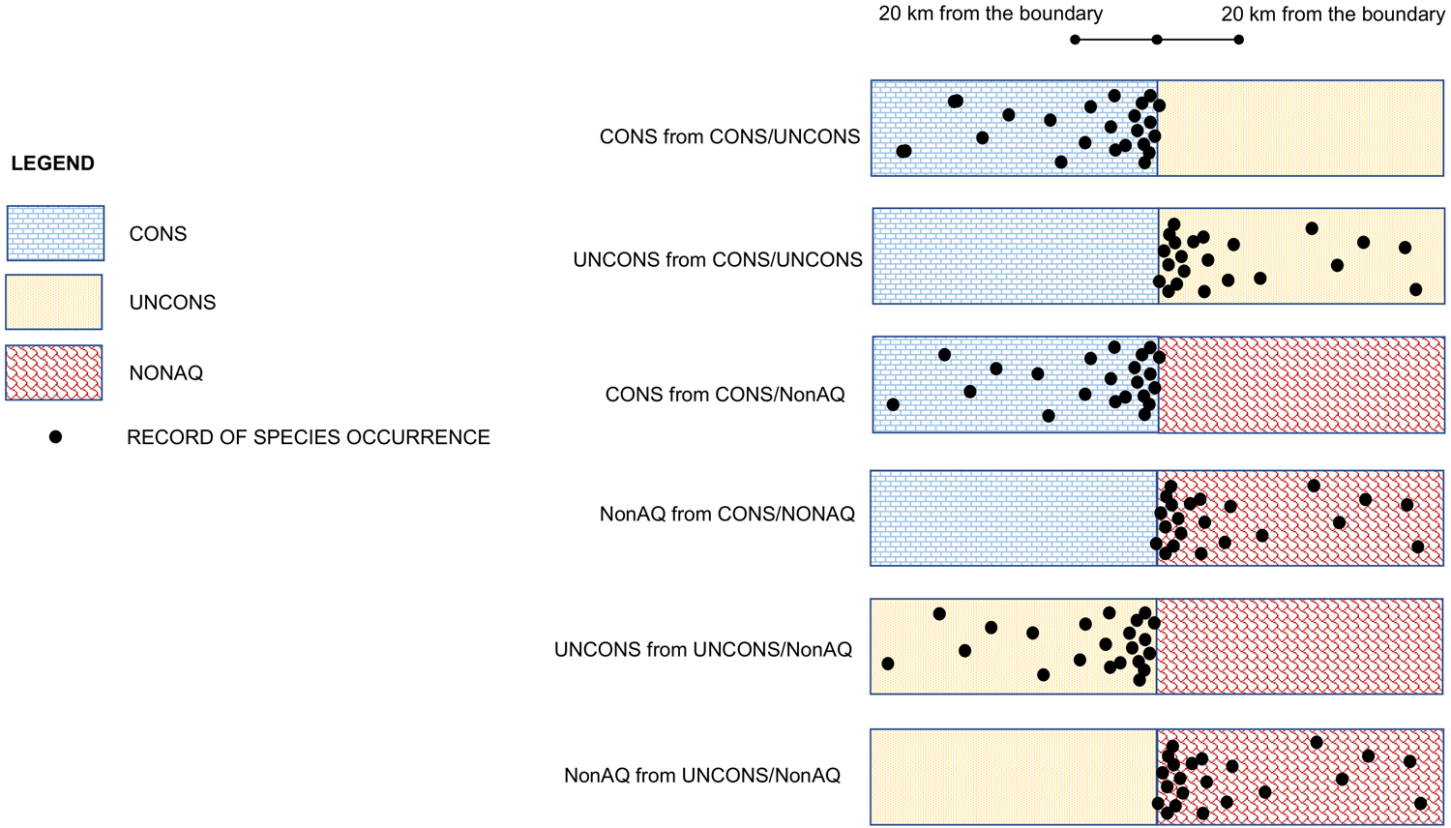
Schematic representation of the rationale of the GIS proximity analysis. Black dots represent the records of occurrence of a given number of stygobitic harpacticoid species within 20 km from the boundary between two adjacent groundwater habitat types. CONS: aquifers in consolidated rocks; UNCONS: aquifers in unconsolidated sediments; NonAQ: practically non-aquiferous rocks^30^. For each pair of groundwater habitats (e.g. CONS/UNCONS) the patterns of occurrence of stygobitic harpacticoids up to 20 km from the boundary were examined first for a habitat (e.g. CONS from CONS/UNCONS) and then for the other (e.g. UNCONS from CONS/UNCONS).

A GIS proximity analysis was conducted in order to assess: (1) the frequency of occurrences of the stygobitic harpacticoid species at the borders between pairs of groundwater habitat types, and (2) the distribution patterns up to 20 km from the boundary (Fig. 3). Twenty kilometres was the distance at which the exponential model functions of the frequency of occurrences plotted against the distances from the boundary reached the asymptotes.

The distances (in m) of the occurrences of the stygobitic harpacticoid species at the boundary of each pair of habitats is provided in the Supplementary Table S1. The analysis was directional, that is we analysed, for each pair of adjacent habitat types, the frequency of occurrences of the species on both sides of the same boundary (Fig. 3). Overall, each habitat type within the context of each pairwise comparison of habitat types was analysed: “CONS from CONS/UNCONS” (distance of species occurring within aquifers in consolidated rocks from the boundary between aquifers in consolidated rocks and aquifers in unconsolidated sediments), “UNCONS from CONS/UNCONS” (distance of species occurring within aquifers in unconsolidated sediments from the boundary between aquifers in consolidated rocks and aquifers in unconsolidated sediments), “NonAQ from CONS/NonAQ” (distance of species occurring within practically non-aquiferous rocks from the boundary between aquifers in consolidated rocks and practically non-aquiferous rocks), “CONS from CONS/NonAQ” (distance of species occurring within aquifers in consolidated rocks from the boundary between aquifers in consolidated rocks and practically non-aquiferous rocks), “NonAQ from UNCONS/NonAQ” (distance of species occurring within practically non-aquiferous rocks from the boundary between aquifers in unconsolidated sediments and practically non-aquiferous rocks), ”UNCONS from UNCONS/NonAQ” (distance of species occurring within aquifers in unconsolidated sediments from the boundary between aquifers in unconsolidated sediments and practically non-aquiferous rocks) (Fig. 3).

The proximity analyses were performed on Euclidean distances, that is using the shortest distance between a record of occurrence to the boundary of two adjacent habitat types in a straight line. The “Euclidean distance” and “Extract values to points” tools in ArcMap 10.0^55^ were used for this purpose. Our starting hypothesis was to observe a rapid decrease in the frequency of occurrences of the stygobitic harpacticoids as the distance from the boundaries increased. This result was expected because (1) the difference in permeability of the geological units at the contact border between two different groundwater habitats types leads to the emergence of ground water and its stygobitic fauna, and (2) the typically low attitude for dispersal of the stygobitic harpacticoids.

We tested the fit of the frequency of occurrences to decreasing exponential models f(x|λ) = λe^−xλ^, where x ≥ 0) as support of this general assumption. For each of the six models, the corresponding R^2^ and residuals were computed. Statistical differences among the six models were investigated by a one-way ANOVA and post-hoc *t*-tests. Prior to ANOVA, the data for each of the six groups were checked for normality using the Kolmogorov–Smirnov test, and homoscedasticity was assessed as well with the Levene’s test with squared deviations, which performs better than other techniques for Type I errors^56^. For both tests, *p* values were set at 0.05. Subsequently, a one-way ANOVA was performed for the six groups, by also assessing the power of the analysis. Post-hoc Holm–Bonferroni tests were performed to compare each pair; *p* values were considered statistically significant if less than the significance level after Holm–Bonferroni correction for multiple comparisons^57^. In order to assess the pattern of the frequency of occurrences of the stygobitic harpacticoids at a smaller spatial scale, the Pyrenean area was selected due to the co-presence of the three groundwater habitat types, being this area also a hotspot of stygobitic harpacticoid biodiversity^13^. All analyses and plots were performed with the statistical package of the NCSS software version 11 for Windows.

## Supplementary information

Supplementary Table.

Supplementary Figures.

## Data Availability

All data used for the analyses are available in Supplementary Materials.
