# Supplementary material for: Spatial distribution of stygobitic crustacean harpacticoids at the boundaries of groundwater habitat types in Europe
Source: Sci Rep. 2020 Nov 4;10:19043. doi: 10.1038/s41598-020-76018-0 (PMC7642423; doi:10.1038/s41598-020-76018-0)
Supplement: Supplementary file 2 — Supplementary Figures. [file 41598_2020_76018_MOESM2_ESM.docx]

**Spatial distribution of stygobitic crustacean harpacticoids at the boundaries of groundwater habitat types in Europe**

Mattia Iannella^1^, Barbara Fiasca^1^, Tiziana Di Lorenzo^2^, Maurizio Biondi^1^, Mattia Di Cicco^1^ and Diana M. P. Galassi^1,*^

^1^University of L’Aquila, Department of Life, Health and Environmental Sciences, Via Vetoio, 67010 Coppito, L’Aquila, Italy

^2^National Research Council, IRET-CNR, Florence, Italy

***** Corresponding author: dianamariapaola.galassi@univaq.it

**Fig. S1.** Normal probability plots built on the residuals of fits (exponential models) for the six groups considered.


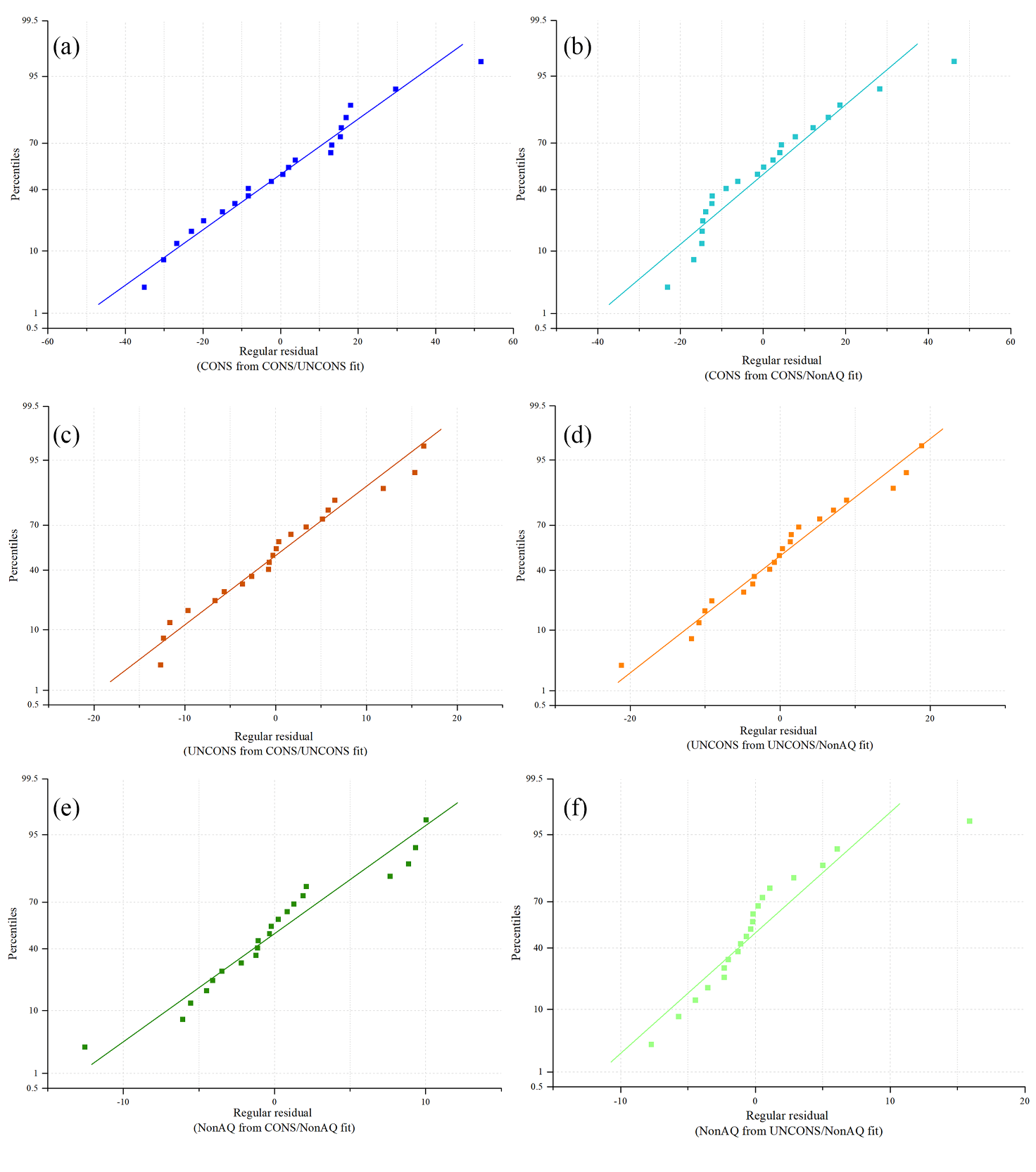


**Fig. S2.** Trends of frequency of occurrences (exponential fit) of the stygobitic harpacticoid copepods at the boundaries between each group of groundwater habitat types in the Pyrenean Region; upper right: boxplots of the same set of data, representing median (black line within the boxplot), mean (square), and min/max values (whiskers) (Abbreviation used: CONS = aquifers in consolidated rocks; UNCONS = aquifers in unconsolidated sediments; NonAQ = practically non-aquiferous rocks).


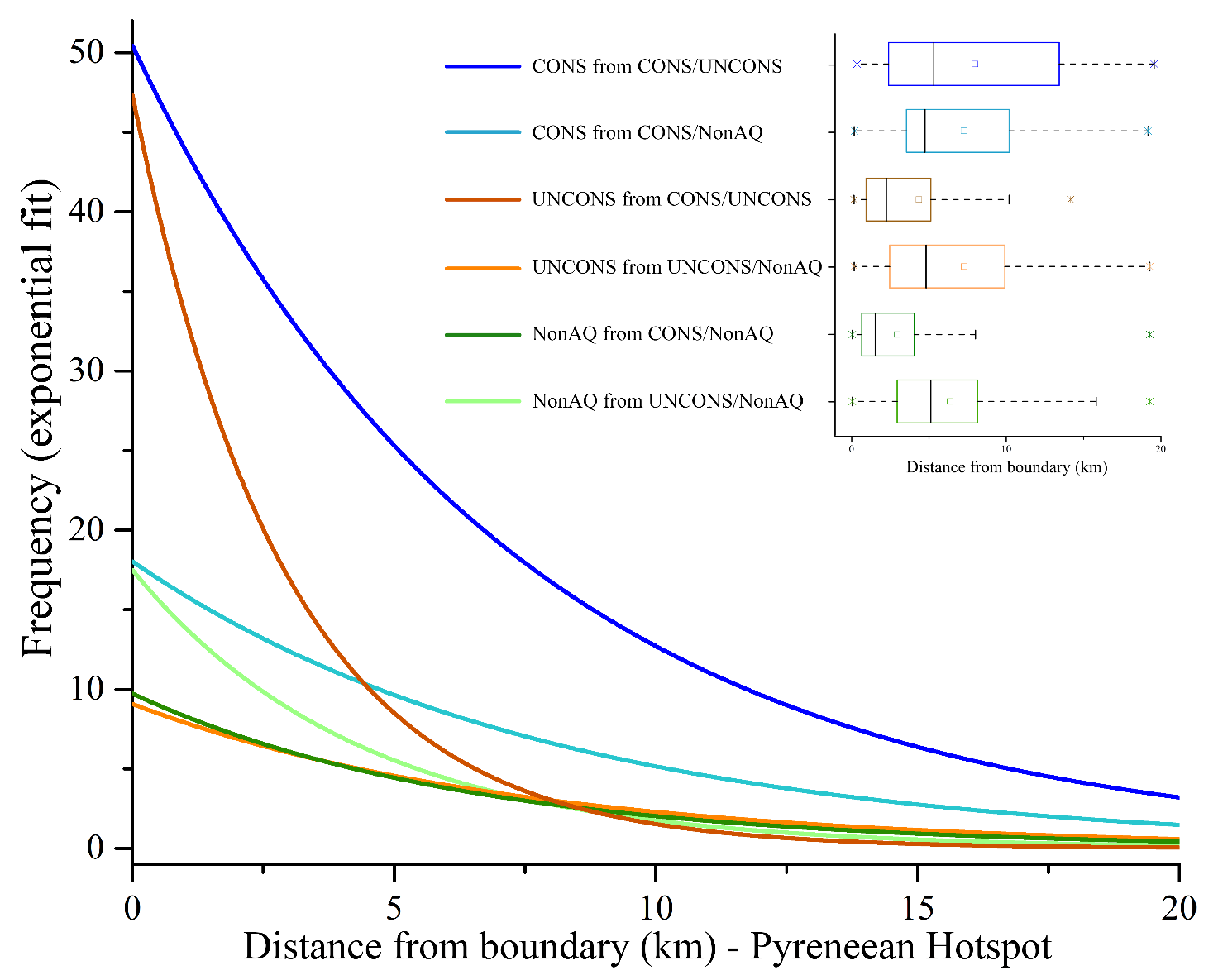


**File S1.** Kolmogorov-Smirnov normality test results for the six groups considered and post-hoc Wilcoxon Rank Sum test resulted as significant among all the possible combinations of pairs of groups, performed in the Pyrenean area.

The six groups did not show a normal distribution (K-S test, *p* = 0.05), with CONS from CONS/UNCONS *p* = 0. 0.011, CONS from CONS/NonAQ *p* = 1.49*10^-10^, UNCONS from CONS/UNCONS *p* = 0.002, UNCONS from UNCONS/NonAQ *p* = 0.024, NonAQ from UNCONS/NonAQ *p* = 0.012, and NonAQ from CONS/NonAQ *p* = 3.31*10^-5^. Considering the non-normality of data, the Kruskal-Wallis test performed to assess the differences among the six groups considered resulted in a statistical difference among them (χ^2^ = 60.72, df = 5, *p* = 8.6*10^-12^). The pairwise Wilcoxon Rank Sum test highlighted statistical differences between the means of nine different pairs of groups, namely: CONS from CONS/UNCONS and UNCONS from CONS/UNCONS (U = 849*, p =* 0.0009), CONS from CONS/UNCONS and NonAQ from CONS/NonAQ (U = 3855*, p =* 4.6199*10^-9^), CONS from CONS/NonAQ and UNCONS from CONS/UNCONS (U = 4863*, p =* 7.9168*10^-5^), CONS from CONS/NonAQ and NonAQ from CONS/NonAQ (U = 9883*, p =* 4.8548*10^-13^), UNCONS from CONS/UNCONS and UNCONS from UNCONS/NonAQ (U = 398*, p =* 0.0076), UNCONS from CONS/UNCONS and NonAQ from UNCONS/NonAQ (U = 419*, p =* 0.0039), UNCONS from CONS/UNCONS and NonAQ from CONS/NonAQ (U = 1670*, p =* 0.0188), UNCONS from CONS/UNCONS and NonAQ from CONS/NonAQ (U = 1762.5*, p =* 3.7746*10^-6^) and NonAQ from UNCONS/NonAQ and NonAQ from CONS/NonAQ (U = 1527.5*, p =* 0.0005).
